# Supplementary material for: Stabilizing a metalloid {Zn12} unit within a polymetallide environment in [K2Zn20Bi16]6−
Source: Nat Commun. 2020 Oct 12;11:5122. doi: 10.1038/s41467-020-18799-6 (PMC7552394; doi:10.1038/s41467-020-18799-6)
Supplement: Supplementary file 1 — Supplementary Information [file 41467_2020_18799_MOESM1_ESM.pdf]

Supplementary Information

Stabilizing a Metalloid $\{Zn_{12}\}$ Unit within a Polymetallide Environment in $[K_2Zn_{20}Bi_{16}]^{6-}$

Eulenstein, Franzke et al.

1. Supplementary Figures

Supplementary Figure 1

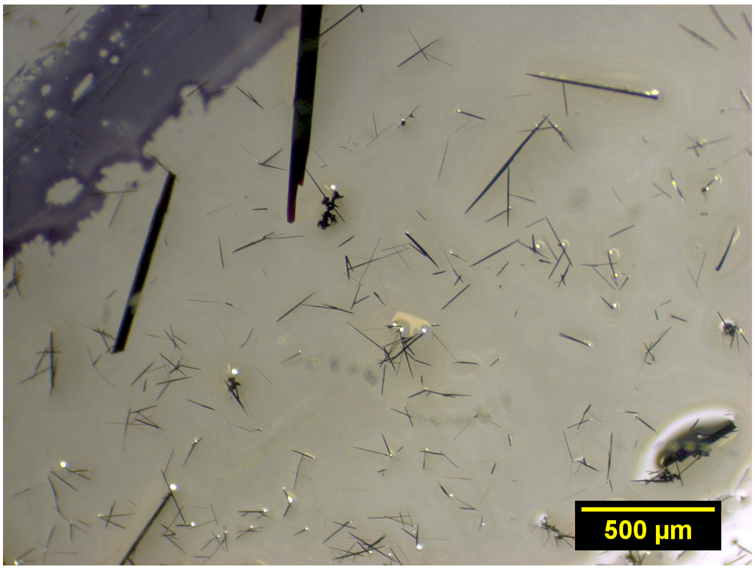

Supplementary Fig. 1 | Light-microscopic image of crystals of compound 1.

Supplementary Figure 2

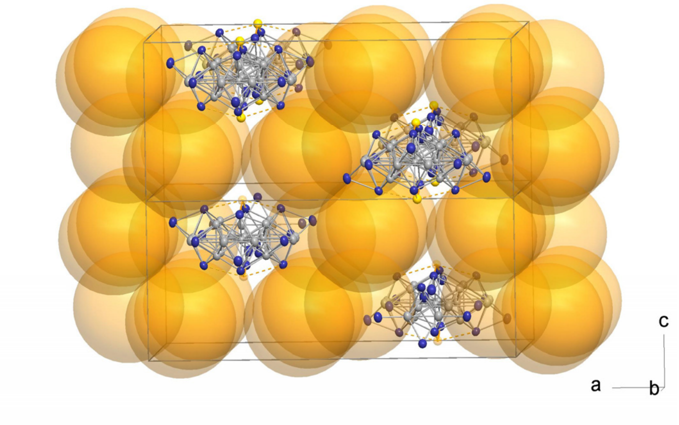

Supplementary Fig. 2 | Two unit cells of the modeled section of the crystal structure of 1. Yellow, semi-transparent spheres with a 5 Å diameter are drawn at the positions of well-refined atoms K3 and K4 to indicate the approximate spatial demand of the $[K(crypt\text{-}222)]^+$ cations, the crypt ligands of which could not be localized from the Fourier map (see **Supplementary Discussion**); the size of the sphere was derived from known crystal structures of compounds with $[K(crypt\text{-}222)]^+$ cations. The $[K_2Bi_{16}Zn_{20}]^{6-}$ anions are in the channels of a distorted honeycomb-like packing of the cations. Each anion is surrounded by 8 cations with closer distances of 4x10.37 and 4x10.43 Å to the center of the inner Zn_4 square, and 10 cations with distances of 12.00 Å (2x) and 12.44 Å (8x).

Supplementary Figure 3

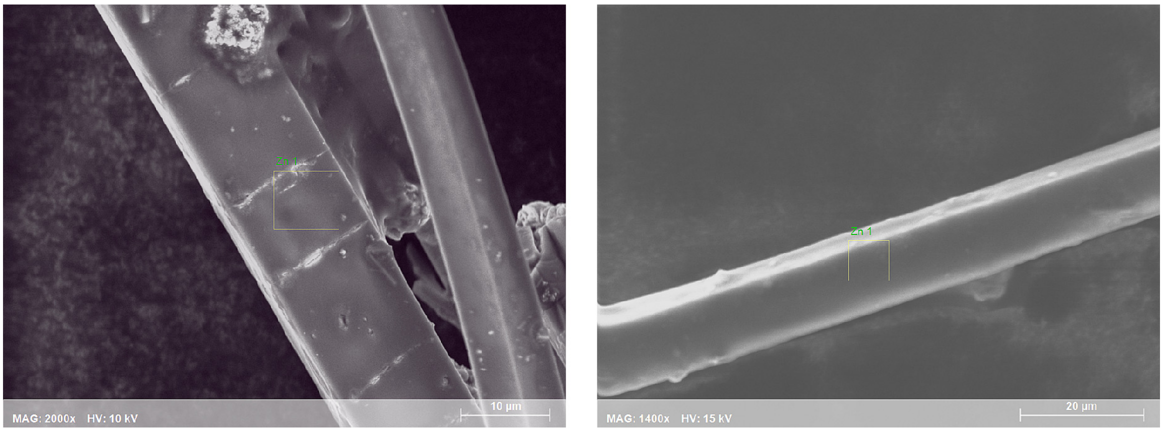

Supplementary Fig. 3 | Images illustrating results of the SEM measurement of freshly mounted single crystals of 1.

Supplementary Figure 4

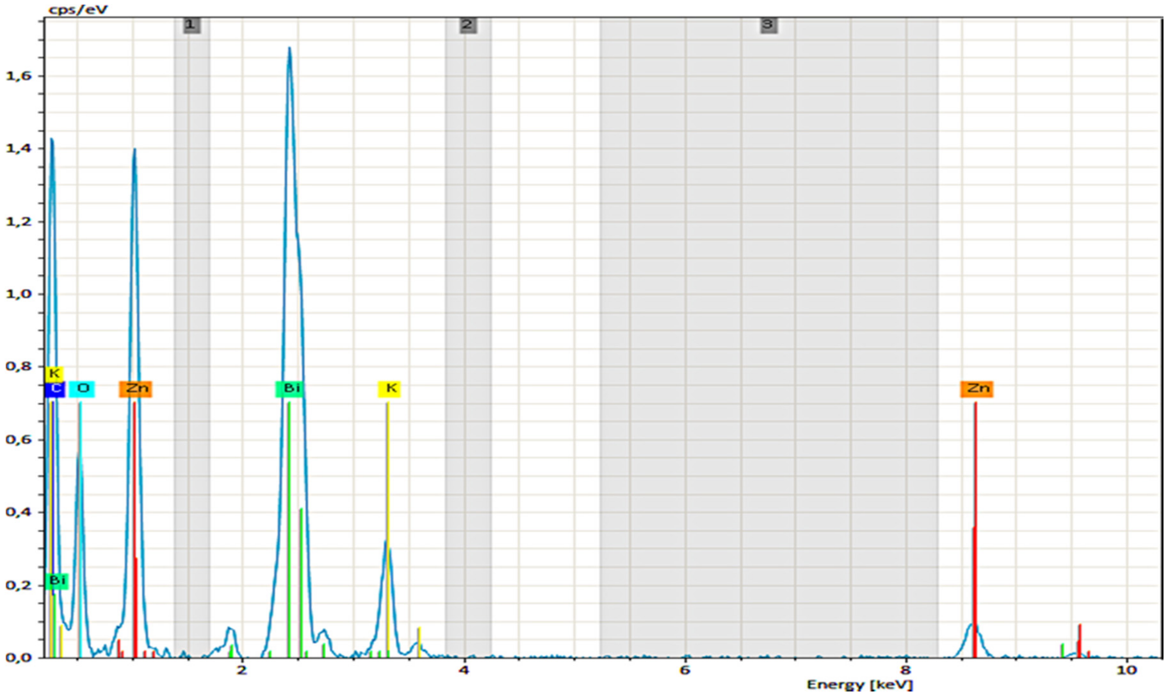

Supplementary Fig. 4 | Energy-dispersive X-ray spectrum of freshly mounted single crystals of 1. Line colors are used as follows: K (yellow), Zn (orange), Bi (green), O (turquoise). The background is indicated by a gray area. The presence of Ga was not detected.

Supplementary Figure 5

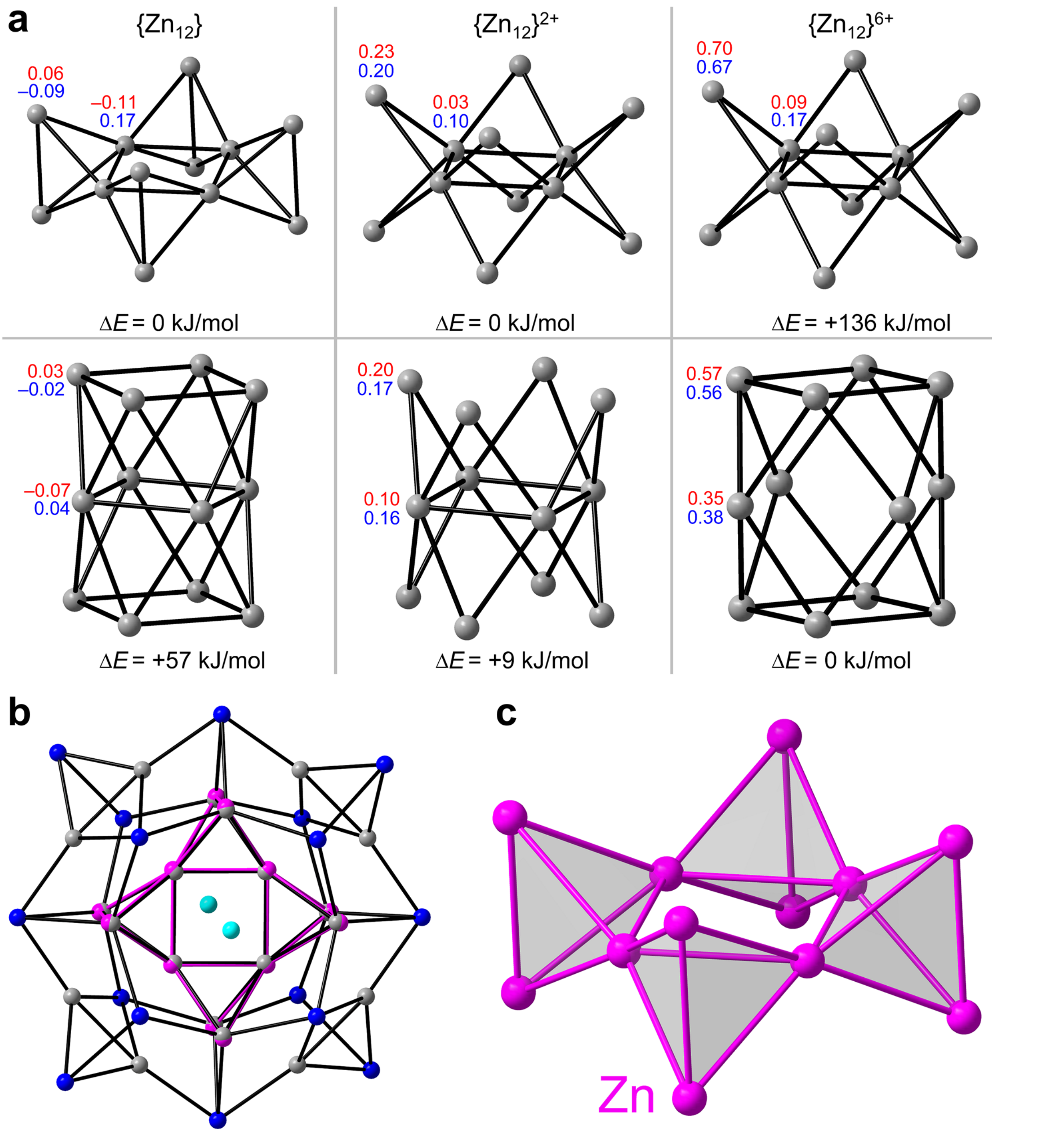

Supplementary Fig. 5 | Quantum chemical study of the inner $\{Zn_{12}\}^q$ unit in 1a. a, Calculated structures, relative energies, and partial charges of two local minimum isomers each of $\{Zn_{12}\}^q$, with q = 0 (left), +2 (center), and +6 (right). The geometry optimizations in D_{2d} symmetry revealed two isomeric local minimum structures for all three charges, with different total energies. Relative energies are given with respect to the more favorable isomer. Partial charges were determined by natural population analyses (NPA, red values) and Mulliken population analyses (blue values). **b**, Overlay of the calculated structure of the entire cluster anion **1a** upon geometry optimization using DFT methods (Zn, grey; Bi, blue; K, turquoise), and the isolated $\{Zn_{12}\}^{\pm 0}$ unit upon geometry optimization using the same methods (Zn, pink). **c**, Calculated structure of the isolated $\{Zn_{12}\}^{\pm 0}$ unit alone.

Supplementary Figure 6

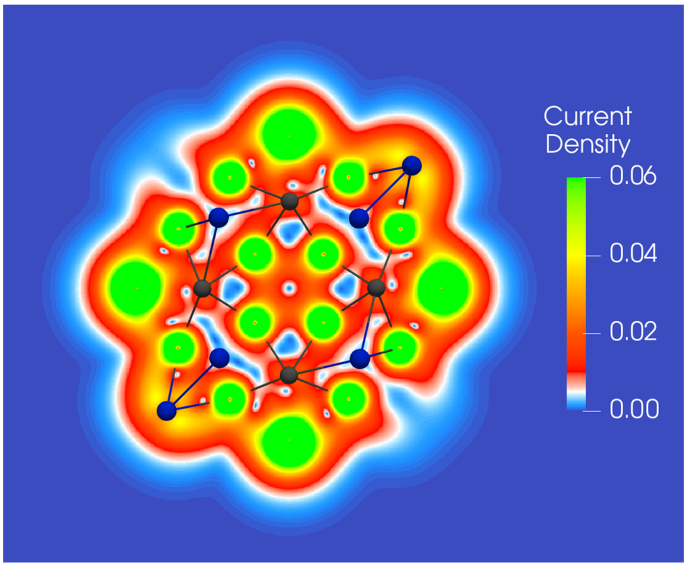

Supplementary Fig. 6 | Magnetically induced current density of $[Zn_{20}Bi_{16}]^{8-}$. The plot shows the magnetically induced current density in a.u., 1 Bohr above the molecular plane at the dhf-TZVP/TPSS level of theory. The corresponding plot 1 Bohr below the plane is identical apart from a rotation by 90°.

Supplementary Figure 7

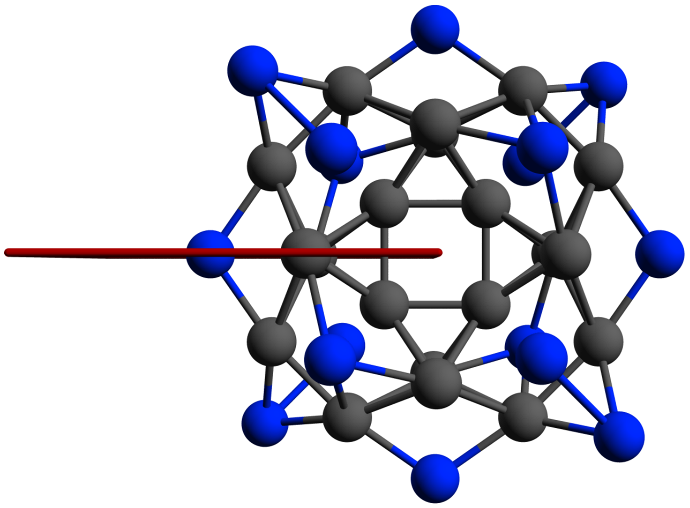

Supplementary Fig. 7 | Integration plane to obtain the current strength based on numerical integration of the magnetically induced current density. The integration plane, which starts at the center of the cluster, is shown in red.

Supplementary Figure 8

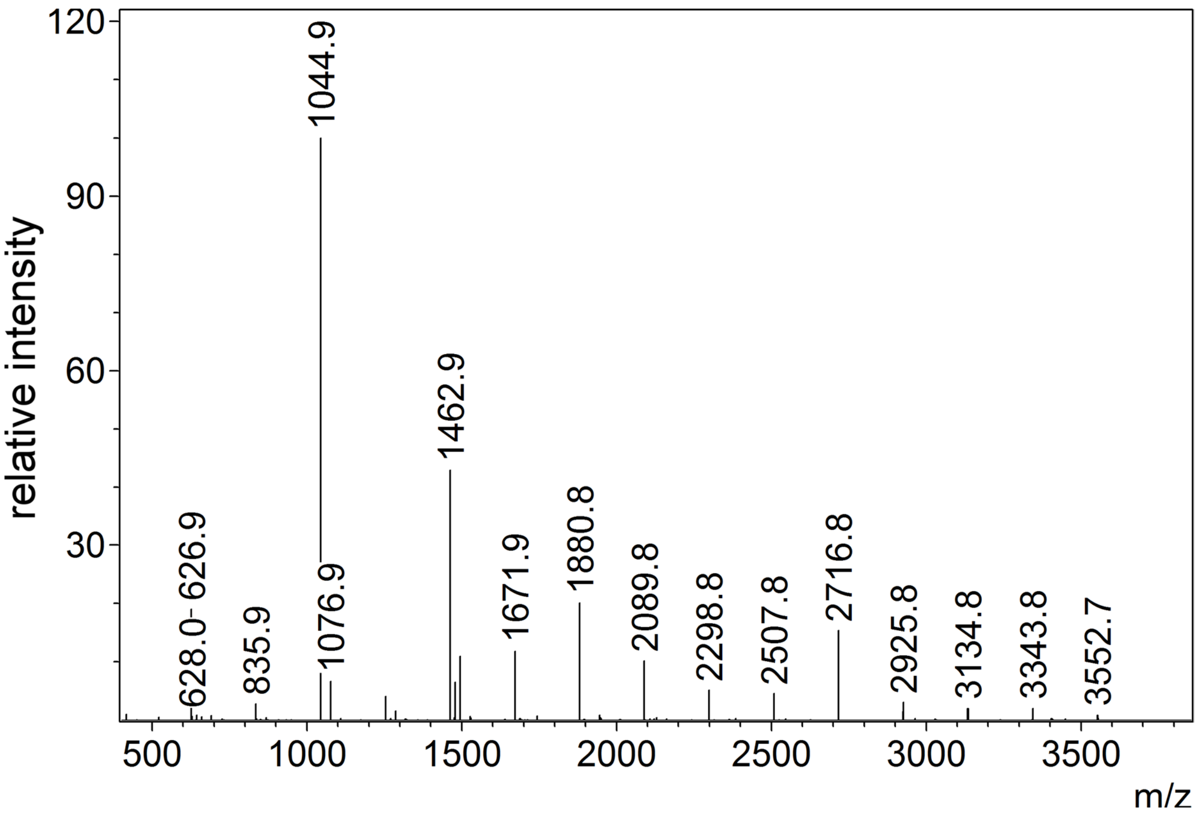

Supplementary Fig. 8 | ESI(−) mass spectrum of a fresh DMF solution of single-crystals of 1. The shown overview spectrum represents the average of data accumulated over the range of 450 – 4000 m/z, where most signals of interest are found. Each labeled peak corresponds to an ion of the composition Bi_x. Signals were found for x = 3-5; 7-17, in addition the protonated species Bi_3H is present (628.0 m/z).

Supplementary Figure 9

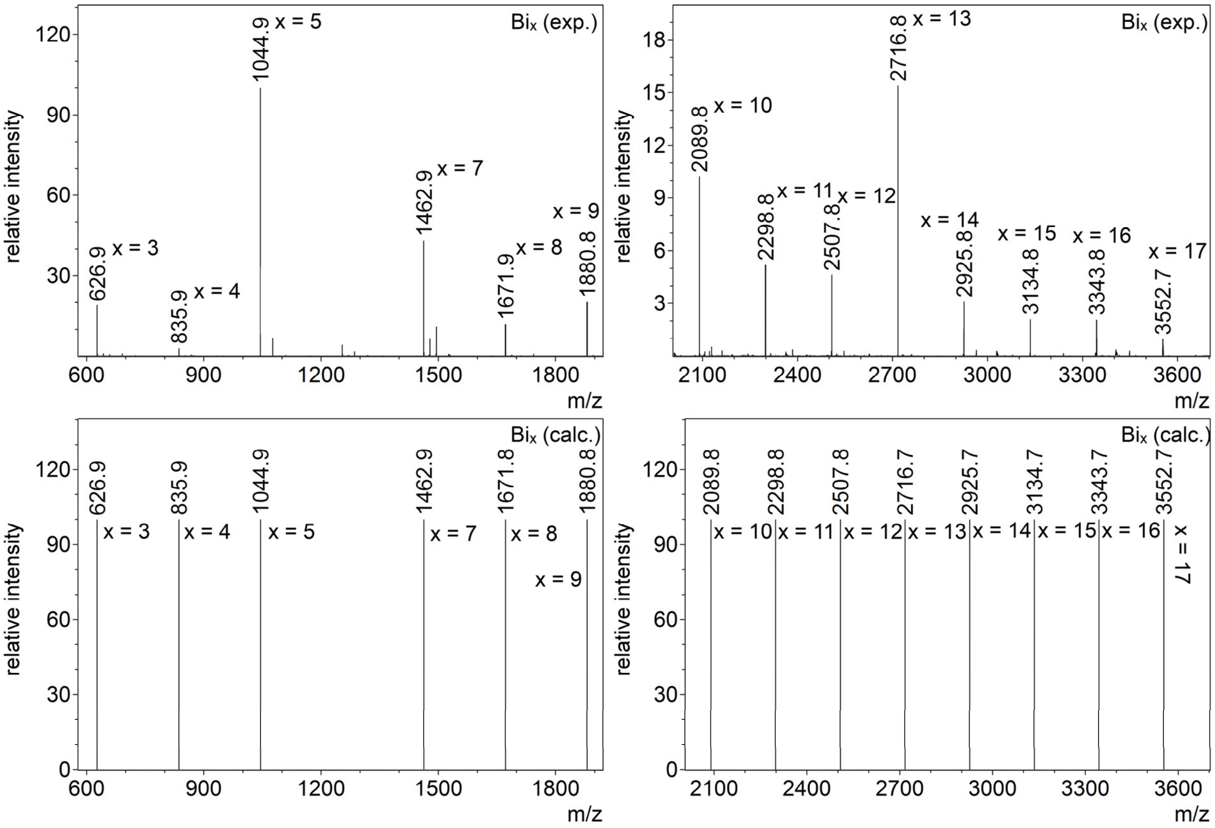

Supplementary Fig. 9 | ESI(−) mass spectra of crystals of 1 dissolved in DMF. The spectra represent details of the spectrum shown in **Supplementary Figure 8**, with a comparison of experimentally observed Bi_x species (top) with calculated ones (bottom).

Supplementary Figure 10

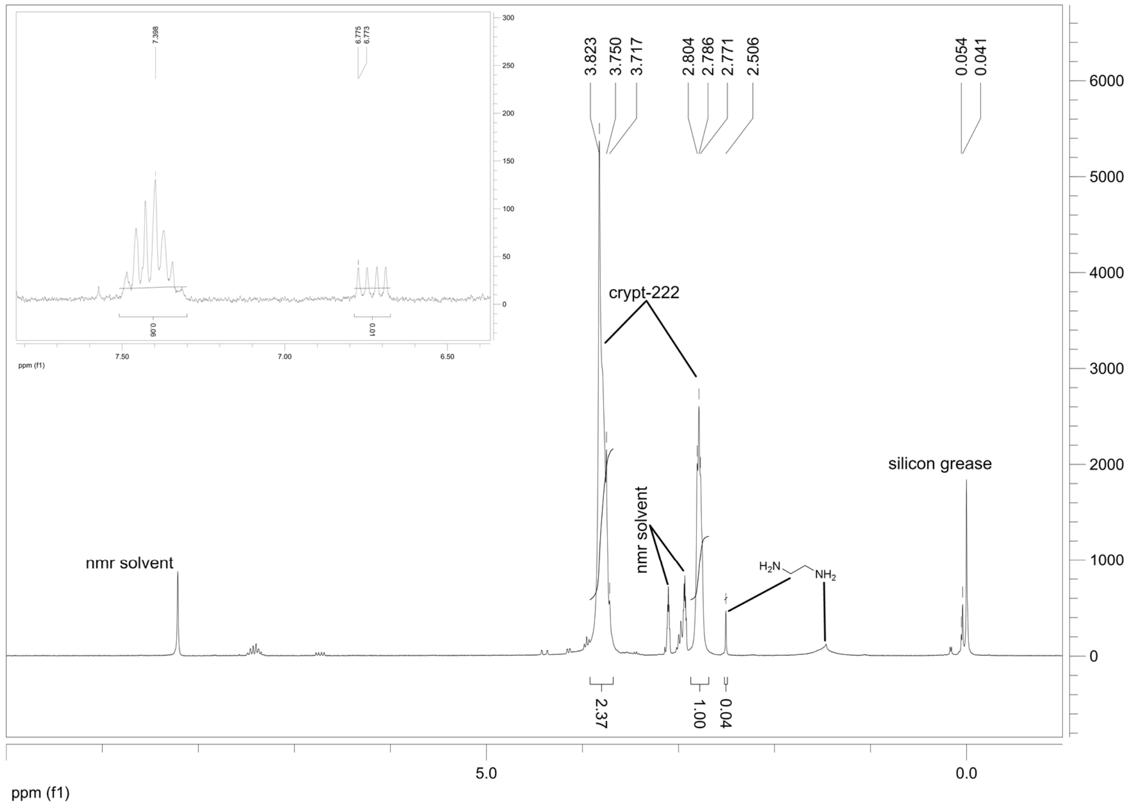

Supplementary Fig. 10 | ¹H-NMR spectrum of a solution of crystals of 1 in DMF-d₇. The spectrum indicates the presence of crypt-222 (as [K(crypt-222)]⁺) and small quantities of crystal solvents en and toluene (see inset), besides silicon grease and NMR solvent.

Supplementary Figure 11

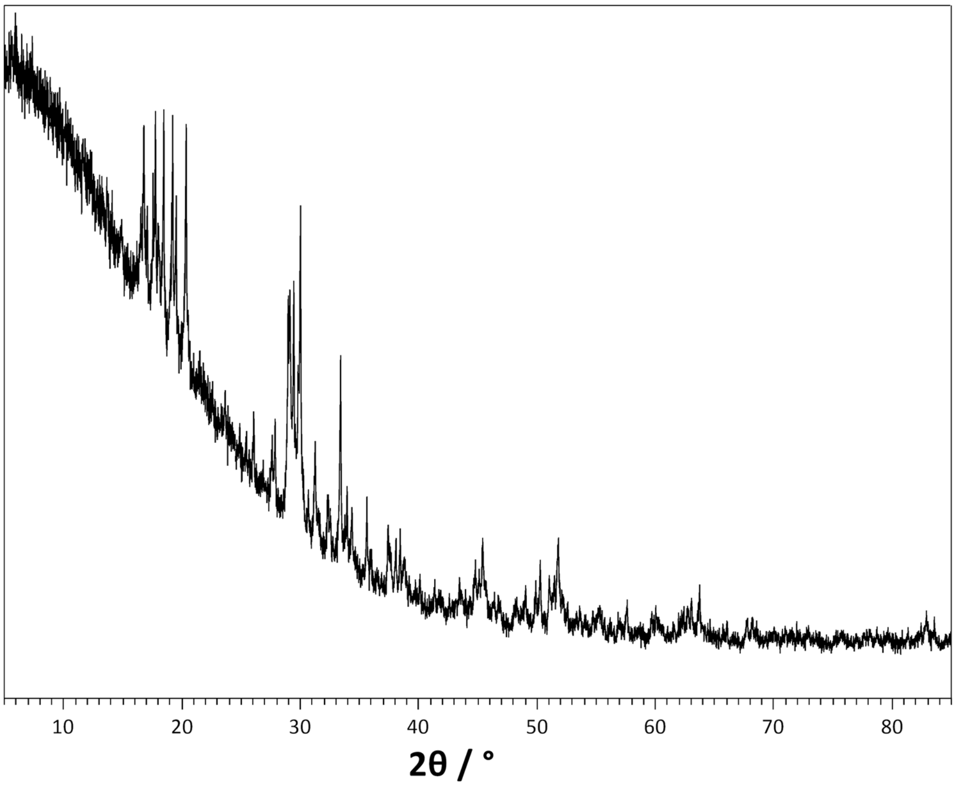

Supplementary Fig. 11 | Powder X-ray diffraction diagram of the ternary mixture of the nominal composition “$K_5Ga_2Bi_4$”.

2. Supplementary Tables

Supplementary Table 1 | Crystal data and details of the structure determination of $[K(crypt\text{-}222)]_6[K_2Zn_{20}Bi_{16}]$ (1).

Empirical formula [a]	$Bi_{16}\ K_8\ Zn_{20}$
Probable formula [b]	$C_{108}\ H_{216}\ Bi_{16}\ K_8\ N_{12}\ O_{36}\ Zn_{20}$
Formula weight [g mol^{-1}] [b]	7257.65
Crystal color, shape	needle, black
Crystal dimensions [mm]	0.27×0.12×0.09
Crystal system	orthorhombic
Space group	*Pmmn*
a [Å]	33.0011(11)
b [Å]	21.5972(9)
c [Å]	14.3006(4)
V [Å^3]	10192.5(6)
Z, ρ_{calc} [g cm^{-3}]	2, 2.365
μ ($Mo_{K\alpha}$) [mm^{-1}] [2)]	30.930
Absorption correction type	Gaussian
2θ range [°]	6.18 – 130.18
Index ranges	$-27>h>38$, $-25>k>25$, $-13>k>16$
Total reflns	65055
Unique reflns [R_{int}]	9119
Obs. reflns [$I > 2\sigma(I)$]	4410
Parameters	117
wR_2 (all data)/ R_1 [$I > 2\sigma(I)$]	0.2170/0.0732
GooF (all data)	1.113
Max peak/hole, [e Å^{-3}]	1.906/–0.948
CCDC number	1969162

[a] Refined model after back-Fourier-transform of the ligand and solvent areas.

[b] With *crypt* ligands, excluding possible *en* and/or *tol* molecules.

Supplementary Table 2 | Results of the energy-dispersive X-Ray spectroscopy (EDS) measurement of freshly mounted single crystals of [K(crypt-222)]$_6$[$K_2Zn_{20}Bi_{16}$] (1).

Element	Element wt. %	Weight % err. (1 σ)	Atom %	Atom ratio obs.	Atom ratio calc.
K – K	7.32	0.21	20.45	9.00	9
Zn – L	27.12	0.80	45.29	19.93	20
Bi – M	65.56	1.70	34.26	15.07	16
Total	99.99		100.00	44	44

Supplementary Table 3 | Total energies (in Hartree) of calculated clusters of the composition $[AZn_{10}E_{16}]^{q-}$. A represents the *two* coordinated cations, and E represents the involved type of pnictogen element.

A<br>E	2 K	2 Ba	2 La	2 Th
Bi	–40218.40274517	–39069.51357442	–39081.52685652	–39833.04812135
Sb	–40627.77070975	–39478.87944342	–39490.88055291	–40242.39192682
As	–72554.09703642	–71405.22707353	–71417.23615558	–72168.74334788

Supplementary Table 4 | Total energies (in Hartree) of calculated clusters of the composition $[AZn_{10}E_{16}]^{q-}$. A represents the *single* coordinated cation, and E represents the involved type of pnictogen element. The sum of energies of 16 (isolated) E atoms are given in the last column. The two rows to the bottom list total energies of crypt-222 complexes ("crypt") and 18-crown-6 complexes ("crown") of the A cations, $[A(crypt\text{-}222)]^{q+}$ or $[A(18\text{-}crown\text{-}6)]^{q+}$.

A / E	K	Ba	La	Th	–	16×A
Bi	–39618.56766523	–39044.12953531	–39050.15728665	–39425.97005899	–39018.71371833	–3432.6365607136
Sb	–40027.93447478	–39453.49566353	–39459.51821675	–39835.32959322	–39428.09195636	–3841.7906642336
As	–71954.25083383	–71379.82506917	–71385.85771728	–71761.67570061	–71354.39540917	–35767.800755712
crypt	–1867.496629061	–1293.069833068	–1299.064035584	–1674.807562576	–1267.592033717	–
crown	–1522.326986764	–947.8909547836	–953.8594387540	–1329.573416891	–922.4439886509	–

Supplementary Table 5 | Reaction energies of the exchange reaction S1. A represents *one or two* coordinated cation(s), and E represents the involved type of pnictogen element. The last column provides corrected numbers upon consideration of the differences in atomization energies (Bi: 207 kJ/mol, Sb 262 kJ/mol, As 302 kJ/mol → $\Delta E_{atom,}$(Bi→Sb) = 16×55 kJ/mol = 880 kJ/mol, $\Delta E_{atom,}$(Bi→As) = 16×95 kJ/mol = 1520 kJ/mol).

E	A	ΔE (kJ/mol)	ΔE considering $\Delta E_{atom,}$(Bi→E) (kJ/mol)
Sb	2 K	–561	319
Sb	2 Ba	–56	324
Sb	2 La	–524	356
Sb	2 Th	–498	382
Sb	K	–558	322
Sb	Ba	–557	323
Sb	La	–543	337
Sb	Th	–-539	341
Sb	–	–599	281
As	2 K	–1392	128
As	2 Ba	–1442	78
As	2 La	–1431	89
As	2 Th	–1394	126
As	K	–1362	158
As	Ba	–1395	125
As	La	–1408	112
As	Th	–1412	108
As	–	–1369	151

Supplementary Table 6 | Reaction energies of the exchange reactions S2 and S3. A represents the type of coordinated cation(s), and E represents the involved type of pnictogen element.

E	A	ΔE_{S2} (kJ/mol)	ΔE_{S3} (kJ/mol)
Sb	K	–3	+31
Sb	Ba	+1	+32
Sb	La	+19	+46
Sb	Th	+41	+50
As	K	–29	−3
As	Ba	–47	–36
As	La	–23	–49
As	Th	+27	–62

Supplementary Table 7 | Reaction energies of the exchange reactions S4 and S5. A represents the type of coordinated cation(s), and E represents the involved type of pnictogen element.

E	A	ΔE_{S4} (kJ/mol)	ΔE_{S5} (kJ/mol)
Bi	K	–182	–132
Bi	Ba	–246	–162
Bi	La	–269	–74
Bi	Th	–360	+107
Sb	K	–179	–163
Sb	Ba	–247	–194
Sb	La	–288	–120
Sb	Th	–402	+58
As	K	–153	–129
As	Ba	–199	–126
As	La	–246	–25
As	Th	–388	+170

Supplementary Table 8 | Reaction energies of the exchange reactions S6 and S7. A represents the type of coordinated cation(s), and E represents the involved type of pnictogen element.

E	A	ΔE_{S6} (kJ/mol)	ΔE_{S7} (kJ/mol)
Bi	K	–126	–76
Bi	Ba	–165	–81
Bi	La	–120	+74
Bi	Th	–134	+333
Sb	K	–123	–106
Sb	Ba	–166	–114
Sb	La	–139	+28
Sb	Th	–176	+284
As	K	–97	–72
As	Ba	–118	–45
As	La	–97	+123
As	Th	–162	+396

Supplementary Table 9 | Coordinates of the calculated molecular structure of $K_2[Zn_{20}Bi_{16}]^{6-}$. The structures were optimized at the DFT level of theory**.** Optimized structures are given in atomic units (Bohr). NICS were calculated at the given coordinates, i.e., the ring centers and the center of mass.

Zn	2.44361619895701	-2.44361619895701	0.02747400345812
Zn	0.02620180442811	-6.02771379772959	-2.60291760229200
Zn	6.02771379772959	-0.02620180442811	-2.60291760229200
Zn	7.86903385283388	-4.22972594221620	0.01695070680266
Zn	4.22972594221620	-7.86903385283388	0.01695070680266
Zn	6.02771379772959	0.02620180442811	2.60291760229200
Zn	-0.02620180442811	-6.02771379772959	2.60291760229200
Zn	2.44361619895701	2.44361619895701	-0.02747400345812
Zn	0.02620180442811	6.02771379772959	2.60291760229200
Zn	7.86903385283388	4.22972594221620	-0.01695070680266
Zn	4.22972594221620	7.86903385283388	-0.01695070680266
Zn	-0.02620180442811	6.02771379772959	-2.60291760229200
Zn	-2.44361619895701	-2.44361619895701	-0.02747400345812
Zn	-4.22972594221620	-7.86903385283388	-0.01695070680266
Zn	-6.02771379772959	0.02620180442811	-2.60291760229200
Zn	-7.86903385283388	-4.22972594221620	-0.01695070680266
Zn	-2.44361619895701	2.44361619895701	0.02747400345812
Zn	-6.02771379772959	-0.02620180442811	2.60291760229200
Zn	-7.86903385283388	4.22972594221620	0.01695070680266
Zn	-4.22972594221620	7.86903385283388	0.01695070680266
Bi	4.70929611087865	-4.70929611087865	-4.99303330267231
Bi	8.47243352567105	-8.47243352567105	-2.85046526705751
Bi	4.79143297521384	-4.79143297521384	4.56630230728159
Bi	4.70929611087865	4.70929611087865	4.99303330267231
Bi	8.47243352567105	8.47243352567105	2.85046526705751
Bi	4.79143297521384	4.79143297521384	-4.56630230728159
Bi	10.82194274929894	0.00000000000000	0.00000000000000
Bi	-4.79143297521384	-4.79143297521384	-4.56630230728159
Bi	-0.00000000000000	-10.82194274929894	0.00000000000000
Bi	-4.70929611087865	-4.70929611087865	4.99303330267231
Bi	-8.47243352567105	-8.47243352567105	2.85046526705751
Bi	-4.70929611087865	4.70929611087865	-4.99303330267231
Bi	-10.82194274929894	0.00000000000000	0.00000000000000
Bi	-4.79143297521384	4.79143297521384	4.56630230728159
Bi	-8.47243352567105	8.47243352567105	-2.85046526705751
Bi	-0.00000000000000	10.82194274929894	0.00000000000000
K	0.00000000000000	0.00000000000000	6.16608131231702
K	0.00000000000000	0.00000000000000	-6.16608131231702
NICS	0.00000000000000	0.00000000000000	0.00000000000000

Supplementary Table 10 | Coordinates of the calculated molecular structure of $[Zn_{20}Bi_{16}]^{8-}$. The structures were optimized at the DFT level of theory**.** Optimized structures are given in atomic units (Bohr). NICS were calculated at the given coordinates, i.e., the ring centers and the center of mass.

Zn	2.44361619895701	-2.44361619895701	0.02747400345812
Zn	0.02620180442811	-6.02771379772959	-2.60291760229200
Zn	6.02771379772959	-0.02620180442811	-2.60291760229200
Zn	7.86903385283388	-4.22972594221620	0.01695070680266
Zn	4.22972594221620	-7.86903385283388	0.01695070680266
Zn	6.02771379772959	0.02620180442811	2.60291760229200
Zn	-0.02620180442811	-6.02771379772959	2.60291760229200
Zn	2.44361619895701	2.44361619895701	-0.02747400345812
Zn	0.02620180442811	6.02771379772959	2.60291760229200
Zn	7.86903385283388	4.22972594221620	-0.01695070680266
Zn	4.22972594221620	7.86903385283388	-0.01695070680266
Zn	-0.02620180442811	6.02771379772959	-2.60291760229200
Zn	-2.44361619895701	-2.44361619895701	-0.02747400345812
Zn	-4.22972594221620	-7.86903385283388	-0.01695070680266
Zn	-6.02771379772959	0.02620180442811	-2.60291760229200
Zn	-7.86903385283388	-4.22972594221620	-0.01695070680266
Zn	-2.44361619895701	2.44361619895701	0.02747400345812
Zn	-6.02771379772959	-0.02620180442811	2.60291760229200
Zn	-7.86903385283388	4.22972594221620	0.01695070680266
Zn	-4.22972594221620	7.86903385283388	0.01695070680266
Bi	4.70929611087865	-4.70929611087865	-4.99303330267231
Bi	8.47243352567105	-8.47243352567105	-2.85046526705751
Bi	4.79143297521384	-4.79143297521384	4.56630230728159
Bi	4.70929611087865	4.70929611087865	4.99303330267231
Bi	8.47243352567105	8.47243352567105	2.85046526705751
Bi	4.79143297521384	4.79143297521384	-4.56630230728159
Bi	10.82194274929894	0.00000000000000	0.00000000000000
Bi	-4.79143297521384	-4.79143297521384	-4.56630230728159
Bi	-0.00000000000000	-10.82194274929894	0.00000000000000
Bi	-4.70929611087865	-4.70929611087865	4.99303330267231
Bi	-8.47243352567105	-8.47243352567105	2.85046526705751
Bi	-4.70929611087865	4.70929611087865	-4.99303330267231
Bi	-10.82194274929894	0.00000000000000	0.00000000000000
Bi	-4.79143297521384	4.79143297521384	4.56630230728159
Bi	-8.47243352567105	8.47243352567105	-2.85046526705751
Bi	-0.00000000000000	10.82194274929894	0.00000000000000
NICS	0.00000000000000	0.00000000000000	0.00000000000000

Supplementary Table 11 | Coordinates of the calculated molecular structure of porphine. The structures were optimized at the DFT level of theory**.** Optimized structures are given in atomic units (Bohr). NICS were calculated at the given coordinates, i.e., the ring centers and the center of mass.

C	8.05700275494477	1.29731120543263	0.00000000000000
C	5.48083502290211	2.13699909472006	0.00000000000000
N	4.00681259841144	0.00000000000000	0.00000000000000
C	5.48083502290211	-2.13699909472006	0.00000000000000
C	8.05700275494477	-1.29731120543263	0.00000000000000
N	0.00000000000000	-3.83334162117032	0.00000000000000
C	-2.05375874305764	-5.40047921491859	0.00000000000000
C	-1.28299402038177	-8.04533577044539	0.00000000000000
C	1.28299402038177	-8.04533577044539	0.00000000000000
C	2.05375874305764	-5.40047921491859	0.00000000000000
C	-4.58062846675022	-4.61372270298630	0.00000000000000
C	-5.48083502290211	-2.13699909472006	0.00000000000000
N	-4.00681259841144	0.00000000000000	0.00000000000000
C	-5.48083502290211	2.13699909472006	0.00000000000000
C	-8.05700275494477	1.29731120543263	0.00000000000000
C	-8.05700275494477	-1.29731120543263	0.00000000000000
C	-4.58062846675022	4.61372270298630	0.00000000000000
C	-2.05375874305764	5.40047921491859	0.00000000000000
N	0.00000000000000	3.83334162117032	0.00000000000000
C	2.05375874305764	5.40047921491859	0.00000000000000
C	1.28299402038177	8.04533577044539	0.00000000000000
C	-1.28299402038177	8.04533577044539	0.00000000000000
C	4.58062846675022	-4.61372270298630	0.00000000000000
C	4.58062846675022	4.61372270298630	0.00000000000000
H	-2.55068245113232	9.65271941419202	0.00000000000000
H	-6.01192811351618	6.08563353025748	0.00000000000000
H	-9.67674628267323	2.54445913907843	0.00000000000000
H	-2.55068245113232	-9.65271941419202	0.00000000000000
H	2.55068245113232	-9.65271941419202	0.00000000000000
H	2.55068245113232	9.65271941419202	0.00000000000000
H	-6.01192811351618	-6.08563353025748	0.00000000000000
H	6.01192811351618	-6.08563353025748	0.00000000000000
H	6.01192811351618	6.08563353025748	0.00000000000000
H	-9.67674628267323	-2.54445913907843	0.00000000000000
H	9.67674628267323	-2.54445913907843	0.00000000000000
H	9.67674628267323	2.54445913907843	0.00000000000000
H	2.08241872937141	0.00000000000000	0.00000000000000
H	-2.08241872937141	0.00000000000000	0.00000000000000
NICS	0.00000000000000	0.00000000000000	0.00000000000000

Supplementary Table 12 | Coordinates of the calculated molecular structure of Zn(II)porphyrine. The structures were optimized at the DFT level of theory**.** Optimized structures are given in atomic units (Bohr). NICS were not calculated for Zn(II) porphyrin.

C	8.03507585153825	1.28964738296147	0.00000000000000
C	5.42486381082624	2.08628970978687	0.00000000000000
N	3.87457171562705	0.00000000000000	0.00000000000000
C	5.42486381082624	-2.08628970978687	0.00000000000000
C	8.03507585153825	-1.28964738296147	0.00000000000000
Zn	0.00000000000000	0.00000000000000	0.00000000000000
N	0.00000000000000	-3.87457171562705	0.00000000000000
C	-2.08628970978687	-5.42486381082624	0.00000000000000
C	-1.28964738296147	-8.03507585153825	0.00000000000000
C	1.28964738296147	-8.03507585153825	0.00000000000000
C	2.08628970978687	-5.42486381082624	0.00000000000000
C	-4.59012602157817	-4.59012602157817	0.00000000000000
C	-5.42486381082624	-2.08628970978687	0.00000000000000
N	-3.87457171562705	0.00000000000000	0.00000000000000
C	-5.42486381082624	2.08628970978687	0.00000000000000
C	-8.03507585153825	1.28964738296147	0.00000000000000
C	-8.03507585153825	-1.28964738296147	0.00000000000000
C	-4.59012602157817	4.59012602157817	0.00000000000000
C	-2.08628970978687	5.42486381082624	0.00000000000000
N	0.00000000000000	3.87457171562705	0.00000000000000
C	2.08628970978687	5.42486381082624	0.00000000000000
C	1.28964738296147	8.03507585153825	0.00000000000000
C	-1.28964738296147	8.03507585153825	0.00000000000000
C	4.59012602157817	-4.59012602157817	0.00000000000000
C	4.59012602157817	4.59012602157817	0.00000000000000
H	-2.55041530443850	9.64671350580613	0.00000000000000
H	-6.04220484235926	6.04220484235926	0.00000000000000
H	-9.64671350580613	2.55041530443850	0.00000000000000
H	-2.55041530443850	-9.64671350580613	0.00000000000000
H	2.55041530443850	-9.64671350580613	0.00000000000000
H	2.55041530443850	9.64671350580613	0.00000000000000
H	-6.04220484235926	-6.04220484235926	0.00000000000000
H	6.04220484235926	-6.04220484235926	0.00000000000000
H	6.04220484235926	6.04220484235926	0.00000000000000
H	-9.64671350580613	-2.55041530443850	0.00000000000000
H	9.64671350580613	-2.55041530443850	0.00000000000000
H	9.64671350580613	2.55041530443850	0.00000000000000

Supplementary Table 13 | Coordinates of the calculated molecular structure of benzene. The structures were optimized at the DFT level of theory**.** Optimized structures are given in atomic units (Bohr). NICS were calculated at the given coordinates, i.e., the ring centers and the center of mass.

C	2.62524979696891	-0.00000000000000	0.00000000000000
C	1.31262489848445	2.27353301545501	0.00000000000000
C	-1.31262489848445	2.27353301545501	0.00000000000000
C	-2.62524979696891	-0.00000000000000	0.00000000000000
C	-1.31262489848445	-2.27353301545501	0.00000000000000
C	1.31262489848445	-2.27353301545501	0.00000000000000
H	4.66241737376797	-0.00000000000000	0.00000000000000
H	2.33120868688399	4.03777188872899	0.00000000000000
H	-2.33120868688399	4.03777188872899	0.00000000000000
H	-4.66241737376797	-0.00000000000000	0.00000000000000
H	-2.33120868688399	-4.03777188872899	0.00000000000000
H	2.33120868688399	-4.03777188872899	0.00000000000000
NICS	0.00000000000000	0.00000000000000	0.00000000000000

Supplementary Table 14 | Coordinates of the calculated molecular structure of $[Hg_8Te_{16}]^{8-}$, taken from Ref. 27. The structures were optimized at the DFT level of theory**.** Optimized structures are given in atomic units (Bohr). NICS were calculated at the given coordinates, i.e., the ring centers and the center of mass.

Hg	7.84476836921105	6.56256236806795	0.08120256411146
Te	12.18349029580723	3.67986309540719	-0.01863092743283
Te	9.22372631166970	11.75460139877972	0.26264584540985
Te	2.82547410054876	5.41825233871588	0.03706204739492
Hg	10.06367293737986	-1.07314845078577	-0.08190095809323
Te	4.64481608249536	14.23536026022344	0.22797986898041
Hg	1.04827212628370	10.24571170217219	0.02842620910373
Te	14.08366135453692	-4.60909887387802	-0.65513839629966
Te	5.27990043357163	-2.92691744341853	0.26940711367885
Te	11.67927404409441	-9.22697010509635	-0.50755039778792
Hg	6.50395790770351	-7.92766665155096	0.12779884853384
Te	3.72588413774941	-12.31887952535333	0.47981084242773
Te	-3.69785851246230	12.36377833549319	-0.17940334876014
Hg	-1.05979977418281	-10.29327990408425	0.23072783077843
Hg	-6.55253767911536	8.00364875629077	-0.08219440896989
Te	-4.64956951674713	-14.27693000226297	-0.03074713759989
Te	-2.83394020373555	-5.46297164742008	0.21217284420988
Te	-11.76358419467505	9.24094794343883	-0.16588303155943
Te	-5.23836895253013	3.02072767726731	0.04811589626802
Te	-9.22605879453778	-11.79374408357056	-0.17317849212397
Hg	-7.84930331847738	-6.60737557202777	0.00565904622272
Te	-14.09965532479717	4.58624009021978	-0.11319317991176
Hg	-9.99577653917460	1.07606146508936	-0.04579854261457
Te	-12.14230666696852	-3.66305871971381	-0.10835850427689
NICS	-0.00014840407298	-0.00034700977032	0.00628771680284

3. Supplementary Discussion

Supplementary Discussion of the X-ray diffraction analyses

The thin, highly-absorbing needles showed very weak diffraction intensities, which rapidly decrease with the diffraction angle. Several attempts have been made on a Bruker Quest device with MoKα radiation, and on a Stoe Stadivari diffractometer using a GeniX 3D CuKα microfocus source. Best data were obtained with the Cu radiation, despite the higher absorption effect. A careful numerical absorption correction was performed based on measured and optimized crystal faces (Stoe XSHAPE). Attempts to improve the crystal quality by using other counterions did not help (as typical in Zintl chemistry); corresponding experiments with ammonium ions, phosphonium ions, alkaline earth cations, and the crown ether 18-crown-6 as a cation sequestering agent instead of crypt-222 failed in the formation of any crystalline material so far.

The structure was solved in space group *Pmmn* with direct methods.[1] The choice of the centrosymmetric space group *Pmmn* seems unquestionable. Solution and refinement in both acentric subgroups, $P2_1mn$ and $Pm2_1n$ ($Pmn2_1$), gave neither significant improvement of the refinement, nor smaller displacement parameters or a resolution of the disorder of the cryptate ligands. The (poor) R_{int} value is approximately the same in the lower monoclinic and triclinic Laue classes. Thus, there is no justification for considering a monoclinic space group.

The heavy atoms of the anion cluster and the K atoms could be localized and refined with anisotropic displacement parameters without any problems.[2] Their unusually large, but reasonable values suppose orientational disorder over close positions in the structure – in accordance with the problems encountered during this analysis. This is most probably the reason why the cryptate ligands at K3 and K4 (on a mirror plane) could not be localized. The size of the region around K3 and K4 and the features of the electron density distribution in Fourier maps are consistent with the assumption of $[K(crypt\text{-}222)]^+$ cations (**Supplementary Figure 2**). Using the PLATON program,[3] the remaining void in the unit cell is calculated to 7218 Å^3, the electron density therein sums up to 2792 electrons. From a structure determination of crypt(222) itself,[4] a molecular volume of 527.8 Å^3 was found; a crypt molecule has 1236 electrons. Thus, six missing crypt ligands in a formula $[K(crypt\text{-}222)]_6[Bi_{16}Zn_{20}K_2]$ at Z = 2 would demand 6333 Å^3 and 2472 electrons per unit cell. The even somewhat larger volume of the void space strongly supports our assumption that K3 and K4 are surrounded by crypt ligands, not by smaller ones like en. Nevertheless, the presence of additional en and/or toluene solvent molecules cannot be excluded. Therefore, when referring to **1**, we use the formula $[K(crypt\text{-}222)]_6[K_2Zn_{20}Bi_{16}]$, keeping in mind that solvent molecules might be present in voids between anions and cations.

In order to reduce impairment of the refinement of the interesting anion cluster by the incomplete model, the influence of these parts was detracted from the data by the back-Fourier-transform method.[3] Thus, the formula of the refined model does not correspond to the real composition of the crystal regarding organic moieties. For completeness, we refer to the most probable formula in **Supplementary Table 1**.

Supplementary Discussion of the Quantum Chemical Investigations of Aromatic Properties of $[K_2Zn_{20}Bi_{16}]^{6-}$ and Induced Current Density in $[Zn_{20}Bi_{16}]^{8-}$

Quantum chemical studies were carried out with TURBOMOLE[5-8] at the DFT/dhf-TZVP[9] level of theory employing scalar-relativistic effective core potentials[10] and the resolution of the identity approximation[11] (RI-*J*). To account for the counter ions and the considerable negative charge, the conductor-like screening model[12,13] (COSMO) was used with default parameters for all except for the cavity of Zn. This was set to 2.223 Å, the default value for both neighboring elements Cu and Ga, as well as for K. After a structure optimization at the dhf-SVP/TPSS/grid m3[14,15] level, the ground state energy was converged up to a threshold of 10^{-7} E_h together with a medium sized grid for the numerical integration of the exchange correlation energy (grid 3).[15] NMR shielding tensors[16,17] were calculated with a threshold of 10^{-6} a.u. for the residual to ensure well-converged perturbed molecular orbitals and the corresponding perturbed density matrix. This is used by GIMIC[18-20] to calculate the magnetically induced current density to quantify the degree of aromaticity based on the magnetic criterion.[20,21] Here, the potassium ions were neglected to simplify the placement of an integration plane. The impact of these ions to the net ring current is expected to be negligible (see below).

A plot of the magnetically induced current density for the $[Zn_{20}Bi_{16}]^{8-}$ cluster is shown in **Supplementary Figure 6**. The cluster sustains a net diatropic ring current of 0.43 to 7.0 nA/T, which is obtained upon integration along a plane starting at the center of the system ranging 10 Bohr beyond the molecule as shown in **Supplementary Figure 7**. The plane is perpendicular to the molecular plane and parallel to the external magnetic field. Due to integration through a heavy atom, only a range of the current strength is given. This ring current suggests weak aromaticity.

The aromaticity was further studied with the nucleus-independent chemical shift (NICS) approach.[22] The NICS values – always calculated at the centers of the molecules − for the $K_2[Zn_{20}Bi_{16}]^{6-}$ and the $[Zn_{20}Bi_{16}]^{8-}$ cluster are −4.2 ppm and −4.4 ppm, respectively, indicating weak aromaticity compared to −8.0 ppm for benzene at the same level of theory. The minor difference in the NICS values between $K_2[Zn_{20}Bi_{16}]^{6-}$ and the $[Zn_{20}Bi_{16}]^{8-}$ justify the above simplification for the current density calculations. Moreover, benzene sustains a net diatropic ring current of 11.5 nA/T at the employed computational methods and the trend of the NICS value is in line with the magnetically induced current density. Zinc porphyrin and porphine sustain a diatropic net ring current of 25.1 nA/T and 25.4 nA/T, respectively. The NICS value of porphine is −14.6 ppm.

Using the scalar-relativistic exact two-component (X2C) approach in its local variant to consider the core electrons,[23-25] reveals the same picture with a NICS value of −3.6 ppm for $K_2[Zn_{20}Bi_{16}]^{6-}$ and −4.2 ppm for $[Zn_{20}Bi_{16}]^{8-}$. Here, we chose the diagonal local approximation to the unitary decoupling transformation (DLU)[26] and the parameters for the finite nucleus model of the scalar and the vector potential were taken from Ref. [27]. The same thresholds as above were employed. However, tailored basis sets (x2c-TZVPall-s)[28] and grids for numerical integration of the exchange-correlation terms were used (grid 3a).[28]

The previously studied topologically porphine-like $[Hg_8Te_{16}]^{8-}$ cluster sustains a diatropic net ring current of 0.24 nA/T and is thus non-aromatic.[29] The corresponding NICS values calculated herein are 1.3 ppm (def2-TZVP)[30] and 1.2 ppm (DLU-X2C/x2c-TZVPall-s). The optimized structure was taken from Ref.

[29], however, the molecule was moved so that the coordinate axes become principal axes of inertia.

Note on the GIMIC code and Python 2:

The used version of GIMIC, see Ref. [19], utilizes Python 3. When using Python 2, the line "from__future__import print_function" must be added to the header of the build version of turbo2gimic.py, which converts TURBOMOLE's (un)perturbed density matrices, structure, and basis set data to the GIMIC input. This allows Python 2.6 and 2.7 to interpret the Python 3 syntax of the print function.

Supplementary Discussion of the Quantum Chemical Investigations of the Cation Coordination in 1a

Exchange reactions were calculated to get insight in the interaction strength between the coordinating pnictogen sites and the coordinated cation in the cluster anion **1a** and modifications of it. **Supplementary Table 3** and **Supplementary Table 4** list the total energies of modified cluster anions with the indicated replacements. The respective reactions that follow from differences of these total energies, according to **Supplementary Equations 1** – **7** are given in **Supplementary Tables 5 – 8**.

Supplementary Table 5 lists the reaction energies of the following replacement reaction (**Supplementary Equation** 1) of the pinctogen atom in the cluster anions with "A" representing *one or two* coordinated cation(s):

$[AZn_{20}Bi_{16}]^{q-} + 16\ E \rightarrow [AZn_{20}E_{16}]^{q-} + 16\ Bi$ (1)

Supplementary Table 6 lists the reaction energies of the following exchange reactions (**Supplementary Equations 2** and **3**), between different clusters:

$[A_2Zn_{20}Bi_{16}]^{p-} + [AZn_{20}E_{16}]^{q-} \rightarrow [AZn_{20}Bi_{16}]^{q-} + [A_2Zn_{20}E_{16}]^{p-}$ (2)

$[AZn_{20}Bi_{16}]^{q-} + [Zn_{20}E_{16}]^{m-} \rightarrow [Zn_{20}Bi_{16}]^{m-} + [AZn_{20}E_{16}]^{q-}$ (3)

Supplementary Table 7 lists the reaction energies of the following exchange reactions (**Supplementary Equations 4** and **5**) between a cluster and crypt-222 (corresponding to equations (1) and (2) in the main document):

$[A_2Zn_{20}Bi_{16}]^{p-}$ + crypt-222 $\rightarrow [AZn_{20}Bi_{16}]^{q-}$ + $[A(\text{crypt-222})]^{n+}$ (4)

$[AZn_{20}Bi_{16}]^{q-}$ + crypt-222 $\rightarrow [Zn_{20}Bi_{16}]^{m-}$ + $[A(\text{crypt-222})]^{n+}$ (5)

Supplementary Table 8 lists the reaction energies of the following exchange reactions (**Supplementary Equations 6** and **7**) between a cluster and 18-crown-6 (corresponding to equations (3) and (4) in the main document):

$[A_2Zn_{20}Bi_{16}]^{p-}$ + 18-crown-6 $\rightarrow [AZn_{20}Bi_{16}]^{q-}$ + $[A(\text{18-crown-6})]^{n+}$ (6)

$[AZn_{20}Bi_{16}]^{q-}$ + 18-crown-6 $\rightarrow [Zn_{20}Bi_{16}]^{m-}$ + $[A(\text{18-crown-6})]^{n+}$ (7)

Supplementary Discussion of the Electrospray Ionization Mass Spectrometry (ESI-MS)

Since the FT-MS device used is only capable of detecting ions with masses of up to 4000 m/z, it was foreseen that the entire cluster anion in **1** would not be detected in the normal monocharged fashion. The detection of anions with higher charges (and corresponding half, third, etc. m/z values) was not excluded, but still not expected owing to the high charge of the original cluster. However, we performed the study to be informed about the fragmentation pattern under ESI-MS conditions.

In agreement with the distinct immiscibility of the atoms in the bulk solids, the cluster seems to segregate into the different element types under ESI-MS conditions. For this, we did not detect any mixed-metallic Zn_xBi_y fragments but observed species with up to 17 Bi atoms instead (see **Supplementary Figure 8**). This indicates that reactive fragments of the cluster are formed in situ, also due to the high negative charge, and seem to stabilize rapidly by aggregation. **Supplementary Figure 9** shows the corresponding sections of the ESI(−) mass spectrum.

Supplementary Discussion of the Identity of the Starting Material

The starting material "'$K_5Ga_2Bi_4$", prepared according to the procedure described in the Methods Section of the main document, was investigated by means of powder X-ray diffraction (see **Supplementary Figure 11**). The diagram indicates poor crystallinity of the sample despite the slow cooling process during preparation. Furthermore, the reflections do not match the diffraction pattern of any known solid comprising the elements K, Ga, and Bi deposited in Pearson's Crystal data (database release 2019/20). The diffraction patterns of the following solids were calculated based on their single-crystal X-ray data (CIFs), and compared with the measured pattern: Bi, α-Ga, KBi, KBi_2, α-KBi_3, β-KBi_3, K_3Bi_2, K_5Bi_4, K_2Ga_3, KGa_3, and K_3Ga_{13}. None of the patterns indicated a structural match.

Moreover, the data were compared with diffraction pattern simulated from the CIFs of the following solids comprising homologous element types, upon replacement of the structure factors of In or Sb with those of Ga or Bi, respectively: $K_5In_2Bi_4$, $K_{10}Ga_{6.33}Sb_3$, K_2GaSb_2, $KGaSb_4$, $KGaSb_2$, or $K_2Ga_2Sb_3$. Again, this procedure did not indicate any structural relationship with "$K_5Ga_2Bi_4$".

The limited data quality of the powder diffraction pattern shown in **Supplementary Figure 11** did not allow for an *ab initio* structure determination and Rietveld refinement.

Supplementary Discussion of the Formation of Compound 1

While the exact processes during the formation of **1** are not yet clarified and subject to current in-depth studies, we suggest the following reaction scheme to be plausible according to the data collected so far (compound **1** and the crystalline by-product $[K(crypt\text{-}222)]_2Bi_4$ given without solvent and crypt for simplification):

10 “$K_5Ga_2Bi_4$” + 20 $ZnPh_2$ $\rightarrow$ $K_8Zn_{20}Bi_{16}$ (**1**) + 40 K^+ + 40 Ph^- + K_2Bi_4 + 20 Ga0↓ + 20 Bi0↓

The reaction scheme reflects and rationalizes the systematically small yield per formula conversion as a consequence of the formation of large amounts of metallic by-products, which we could confirm to form in an approximately 1:1 ratio, and which we plan to recycle for the formation of new reactant as they are easily separable from the reaction mixture. The formation of Ph^-, and a potential follow-up reaction with the solvent en to form benzene and $(H_2NCH_2CH_2NH)^-$, are known for reactions of Zintl anions with metal phenyl compounds.[31]

4. References for the Supplementary Information

[1] Sheldrick, G. M. SHELXT – Integrated space-group and crystal-structure determination. *Acta Crystallogr., Sect. A: Found. Adv.* **71,** 3-8 (2015).

[2] Sheldrick, G. M. Crystal structure refinement with SHELXL. *Acta Crystallogr., Sect. C: Struct. Chem*. **71,** 3-8 (2015).

[3] Spek, A. L. PLATON SQUEEZE: a tool for the calculation of the disordered solvent contribution to the calculated structure factors. *Acta Crystallogr., Sect. C: Struct. Chem*. **71,** 9-18 (2015).

[4] Metz, B., Moras, D. & Weiss, R., Polyoxamacrobicyclic diamines. Structure of 4,7,13,16,21,24-hexaoxa-1,10-diazabicyclo[8.8.8]hexacosane and of its bisborohydride, $C_{18}H_{36}N_2O_6{\cdot}2BH_3$, *J. Chem. Soc., Perkin Trans.* **2,** 423-429 (1976).

[5] TURBOMOLE 7.4.1 2019 & TURBOMOLE 7.5 2020. TURBOMOLE is a Development of University of Karlsruhe and Forschungszentrum Karlsruhe, 1989–2007, TURBOMOLE GmbH since 2007 (Accessed 3 March 2020); https://www.turbomole.org.

[6] Ahlrichs, R., Bär, M., Häser, M., Horn, H. & Kölmel, C. Electronic structure calculations on workstation computers: The program system turbomole. *Chem. Phys. Lett.* **162,** 165-169 (1989).

[7] Furche, F., Ahlrichs, R., Hättig, C., Klopper, W., Sierka, M. & Weigend, F. Turbomole. *Wiley Interdiscip. Rev.: Comput. Mol. Sci.* **4,** 91-100 (2014).

[8] Balasubramani, S. G. et al. TURBOMOLE: Modular program suite for *ab initio* quantum-chemical and condensed-matter simulations. *J. Chem. Phys.* **152,** 184107 (2020).

[9] Weigend, F. & Baldes, A. Segmented contracted basis sets for one- and two-component Dirac-Fock effective core potentials. *J. Chem. Phys.* **133,** 174102 (2010).

[10] Metz, B., Stoll & H., Dolg, M. Small-core multiconfiguration-Dirac–Hartree–Fock-adjusted pseudopotentials for post-d main group elements: Application to PbH and PbO. *J. Chem. Phys.* **113,** 2563 (2000).

[11] Weigend, F. Accurate Coulomb-fitting basis sets for H to Rn. *Phys. Chem. Chem. Phys.* **8,** 1057-1065 (2006).

[12] Schäfer, A., Klamt, A., Sattel, D., Lohrenz, J. C. W. & Eckert, F. COSMO Implementation in TURBOMOLE: Extension of an efficient quantum chemical code towards liquid systems. *Phys. Chem. Chem. Phys*. **2,** 2187-2193 (2000).

[13] Klamt, A. & Schüürmann, G. COSMO: a new approach to dielectric screening in solvents with explicit expressions for the screening energy and its gradient. *J. Chem. Soc., Perkin Trans*. **2**, 799-805, (1993).

[14] Tao, J., Perdew, J. P., Staroverov, V. N. & Scuseria, G. E. Climbing the Density Functional Ladder: Nonempirical Meta–Generalized Gradient Approximation Designed for Molecules and Solids. *Phys. Rev. Lett.* **91,** 146401 (2003).

[15] Treutler, O. & Ahlrichs, R. Efficient molecular integration schemes. *J. Chem. Phys.* **102,** 346-354 (1995).

[16] Reiter, K., Mack, F. & Weigend, F. Calculation of Magnetic Shielding Constants with meta-GGA Functionals Employing the Multipole-Accelerated Resolution of the Identity: Implementation and Assessment of Accuracy and Efficiency. *J. Chem. Theory Comput*. **14,** 191-197 (2018).

[17] Reiter, K., Kühn, M. & Weigend, F. Vibrational circular dichroism spectra for large molecules and molecules with heavy elements. *J. Chem. Phys.* **156,** 054102 (2017).

[18] Jusélius, J., Sundholm, D. & Gauss, J. Calculation of current densities using gauge-including atomic orbitals. *J. Chem. Phys*. **121,** 3952-3963 (2004).

[19] GIMIC version 2.1.4 (3a5f0eb) 2019, available via https://github.com/qmcurrents/gimic (Accessed March 3 2020).

[20] Fliegl, H., Taubert, S., Lehtonen, O. & Sundholm, D. The gauge including magnetically induced current method. *Phys. Chem. Chem. Phys*. **13,** 20500-20518 (2011).

[21] Sundholm, D., Fliegl, H. & Berger, R. J. Calculations of magnetically induced current densities: theory and applications. *Wiley Interdiscip. Rev.: Comput. Mol. Sci.* **6,** 639-678 (2016).

[22] Schleyer, P. v. R., Maerker, C., Dransfeld, A., Jiao, H. & van Eikema Hommes, N. J. R. Nucleus-Independent Chemical Shifts: A Simple and Efficient Aromaticity Probe, *J. Am. Chem. Soc.* **118,** 6317-6318 (1996).

[23] Peng, D., Middendorf, N., Weigend, F. & Reiher, M. An efficient implementation of two-component relativistic exact-decoupling methods for large molecules. *J. Chem. Phys*. **138,** 184105 (2013).

[24] Franzke, Y. J., Middendorf, N. & Weigend, F. Efficient implementation of one- and two-component analytical energy gradients in exact two-component theory. *J. Chem. Phys*. **148,** 104110 (2018).

[25] Franzke, Y. J. & Weigend, F. NMR Shielding Tensors and Chemical Shifts in Scalar-Relativistic Local Exact Two-Component Theory. *J. Chem. Theory Comput.* **15,** 1028-1043 (2019).

[26] Peng, D. & Reiher, M. Local relativistic exact decoupling. *J. Chem. Phys*. **136,** 244108 (2012).

[27] Visscher, L. & Dyall, K. G. DIRAC–FOCK ATOMIC ELECTRONIC STRUCTURE CALCULATIONS USING DIFFERENT NUCLEAR CHARGE DISTRIBUTIONS. *At. Data Nucl. Data Tables* **67,** 207-224 (1997).

[28] Franzke, Y. J., Treß, R., Pazdera, T. M. & Weigend, F. Error-consistent segmented contracted all-electron relativistic basis sets of double- and triple-zeta quality for NMR shielding constants. *Phys. Chem. Chem. Phys*. **21,** 16658-16664 (2019).

[29] Donsbach, C., Reiter, K., Sundholm, D., Weigend, F. & Dehnen, S. $[Hg_4Te_8(Te_2)_4]^{8-}$: A Heavy Metal Porphyrinoid Embedded in a Lamellar Structure. *Angew. Chem. Int. Ed*. **57,** 8770-8774 (2018).

[30] Weigend, F. & Ahlrichs, R. Balanced basis sets of split valence, triple zeta valence and quadruple zeta valence quality for H to Rn: Design and assessment of accuracy. *Phys. Chem. Chem. Phys.* **7,** 3297-3305 (2005).

[31] Ugrinov, A. & Sevov, S. C. Derivatization of Deltahedral Zintl Ions by Nucleophilic Addition: $[Ph{-}Ge_9{-}SbPh_2]^{2-}$ and $[Ph_2Sb{-}Ge_9{-}Ge_9{-}SbPh_2]^{4-}$. *J. Am. Chem. Soc.* **125,** 14059-14064 (2003).
